# Supplementary material for: Patterns of Intron Gain and Loss in Fungi
Source: PLoS Biol. 2004 Nov 30;2(12):e422. doi: 10.1371/journal.pbio.0020422 (PMC532390; doi:10.1371/journal.pbio.0020422)
Supplement: Table S1 — Also available at http://genes.mit.edu/NielsenEtAl/. (4.3 MB ZIP). [file pbio.0020422.st001.zip › NielsenEtAl/html/756.html]

AN6985.1.NCU01701.1.MG08591.1.FG01430.1


```
 CLUSTAL W (1.82) Multiple Sequence Alignments - Introns Inserted


Sequence 1: NCU01701.1	623 aa
Sequence 2: MG08591.1	636 aa
Sequence 3: FG01430.1	610 aa
Sequence 4: AN6985.1	615 aa
Alignment Length: 658 aa
Number Identitical Residues: 340 aa
Alignment Score (without introns) 16207


MG08591.1 	MDIGPVNFCSLVFFFACFLAK2LCQSMPPLSVC2LA1NRVDRILLVSSRALKST0EHYIG
NCU01701.1	---------------------~MTPEQVFTGSG~NS~RRFSTIREQITYRRVAQ~DHYIG
FG01430.1 	---------------------~-----------~--~---------MDRPPQIL0DHYIG
AN6985.1  	--------------------M~ASVALPFHRPM~GK~ASRQASLDTRDEILLQA~DHFIG
          	                                        .              :*:**

MG08591.1 	IDVGTGSARACLIDVTGDIKSLATKEIKLWQPAAGYE~GSHY0EQSTTDIWDAICFCVKK
NCU01701.1	IDVGTGSARACIIDSTGDIKALAAQNIKLWTPSSGLE~GSQY0EQSTTDIWNAICNCVKQ
FG01430.1 	VDVGTGSARACIIDETGDIKALASENIKLWQPETGY-~---Y0EQSTTDIWQCICECVRR
AN6985.1  	IDVGTGSARACIIDAKGDIVGLASENIGLWQPQTGYY0-AFQ~EQSTTDIWRCICVAVQR
          	:**********:** .*** .**:::* ** * :*    :   ******** .** .*::

MG08591.1 	VVTDTGIDTSTIHGLGFDATCSLAVFSHDTDEPIPVTGPDFSNASGEDRNVILWLDHRPL
NCU01701.1	VLAESKVDPNSVKGIGFDATCSLAVFTHDTNEPVPVTGPDFKND-GNDRNVILWLDHRPL
FG01430.1 	VVSESTVNPSSIKGIGFDATCSLAVFTHDTDEPLPVTGPDFTND-GNDRNVILWLDHRPV
AN6985.1  	AINQHNIDPETVRGIGFDATCSLAVFSNVTDEPISVTGPNFDTD----RNVILWLDHRPV
          	.: :  ::..:::*:***********:: *:**:.****:* .     ***********:

MG08591.1 	AEAEKINATGHNLLRYVGGKMSVEMEIPKILWLKNNMPKDLFDRCKFYDLADALTHLATG
NCU01701.1	AEAEKINNTNHNLLRYVGGKMSVEMEIPKVLWLKNNMPPELFARCKFFDLTDALTYIATG
FG01430.1 	EETEKINNTNHKLLKYVGGKMSIEMEMPKVLWLKNHMPPEVFERAKFYDLADALTHLATG
AN6985.1  	EEAEKINATNHNLLRYVGGKMSVEMEIPKVLWLKNNMPKELFADCKFYDLADALTHIATG
          	 *:**** *.*:**:*******:***:**:*****:** ::*  .**:**:****::***

MG08591.1 	GNTRSFCSTVCKQGFVPVGVDGSVKGWQEDFYHTIGLGDLVEDDFKRMGGVNGV0SGEYL
NCU01701.1	KEIRSFCSTVCKQGFVPVGVDGSVKGWQEDFYETIGLGDLVTDNFIRMGGVDGV0NGSWM
FG01430.1 	NETRSYCSVVCKQGYVPVGVDGSVKGWQEDFYHDIGLGDLTKDNFKQMGGVNGV0NGTYV
AN6985.1  	NEKRSYCSVICKQGFVPVGVDGSVKGWQEDFLVQIGLKDLTEDNFKRMGGVDGV0NGDYL
          	 : **:**.:****:****************   *** **. *:* :****:** .* ::

MG08591.1 	SAGELVGGLSDKAATELGLRPGIAVGSGVIDAYAGWIGTVGAKVNLTPDELDETVAPNDV
NCU01701.1	SAGELVGGLSEEAGQQLGLPAGIAIGSGVIDAYAGWIGTVGAKVKLSPDHRDDSVAPNDV
FG01430.1 	SAGEPVGTLSRLAANQLGLPMGIPVGSGVIDAYAGWIGTVGAKVDLGDDELNAAVPHNDL
AN6985.1  	SAGELVGKLCEKAAADLGLPAGIAIGSGVIDAYAGWIGTVGAKVELESEQLTSDAAKNDI
          	**** ** *.  *. :***  **.:*******************.*  :.    .. **:

MG08591.1 	AQAFTRLAAVAGTSTCHLAMSKEAVFVDGVWGPYRDVLIPGFWMAEGGQSATGELMRHMI
NCU01701.1	SQAFTRLAAVAGTSTCHLAMSKGPVFVPGVWGPYRDVLIPGYWMAEGGQSATGELMKHML
FG01430.1 	AQAFTRLAAVAGTSTCHLALSKEPVFVPGVWGPYRDVLLPEFWMAEGGQSATGELLRHML
AN6985.1  	TQAFSRLAAVAGTSTCHLAMSPEPVFVPGVWGPYRDTIIPGFWMAEGGQSATGELLKHVI
          	:***:**************:*  .*** ********.::* :*************::*::

MG08591.1 	ETHAAFVETQAAAVAQGKNIYDYLNEHLRQMASKVQAPSISWLARHFFFYGDLWGNRSPV
NCU01701.1	ETHVAYDETVKEAEAAGKNIYDYLNEHLKEMAKKTNAPSISYLVRHFFFYGDLWGNRSPI
FG01430.1 	DIHPAYNETCALAKAEDKHIYDFLNTHLELMKEKHNAPSISYLGRHHFFYGDLWGNRSPI
AN6985.1  	ETHPAYNQALSVAESYNTNIYEYLNEHLREMMTEQKAPSISYMARHIFFYGDLWGNRSPI
          	: * *: ::   * : ..:**::** **. *  : :*****:: ** ************:

MG08591.1 	ADPNMRGAIVGMSSDKTADSLALLYYSTMEFIALQTRQIVETMNKAGHSISSIFMSGSQC
NCU01701.1	ADPNMRGAIIGMSNDKSKDGMALLYYSTMEFIALQTRQIVEAMNTAGHTIKSIFMSGSQC
FG01430.1 	ADPNMKGSMIGLDSDKSTDNMALWYYATMEFIAMQTRQIVEQMNNSGHEISSIFMSGSQC
AN6985.1  	ADPNMAGSIIGLTSDQSVDGLAIYYYATLEFIALQTRQIVETMNKSGHSIASIFMSGSQC
          	***** *:::*: .*:: *.:*: **:*:****:******* **.:** * *********

MG08591.1 	QNEILMDLIATACRMHVLIPRYVNAAVVHGAAMLGAKAASADAEGKTED--LWSIMDRMS
NCU01701.1	QNEILMDLIATACDMPVLIPRYVNAAVVHGAAMLGAKAASAKPDGTTEP--LWDIMDRMS
FG01430.1 	QNPVLMNLLATACGMPVLIPRYVNAAVVHGAAMLGAKAASHSKEDGSEPESLWSIMDRMS
AN6985.1  	QNDILVKLIASACGMPVLIPRYIHAAVAIGAAMLGAKAASADPEGNTED--LWSIMDRMS
          	** :*:.*:*:** * ******::***. *********** . :. :*   **.******

MG08591.1 	KPGRLVNPAQDQALSKLLDAKYEIFLEQCRSQQEYRRQVDKAIGA---------------
NCU01701.1	KPGKTVWSRGDPAEKKLLDAKYEIFLDQARTQQEYRKKIDAALKG--SSLEGQEAN----
FG01430.1 	KRGRLVDPSTNEDEKTLLDAKYEIFLDMCKTQQEYRSKINKATASKKSRMNGTVNGTVNG
AN6985.1  	KPGRRIVPTDNENEKALLEVKYKVFLEQCYQQKQYRALVDEAVNSWKLS-----------
          	* *: : .  :   . **:.**::**: .  *::**  :: *  . .             

MG08591.1 	-----
NCU01701.1	-----
FG01430.1 	TVNGA
AN6985.1  	-----
          	
```
